# Supplementary material for: Critical angle reflection imaging for quantification of molecular interactions on glass surface
Source: Nat Commun. 2021 Jun 7;12:3365. doi: 10.1038/s41467-021-23730-8 (PMC8185113; doi:10.1038/s41467-021-23730-8)
Supplement: Supplementary file 1 — Supplementary information [file 41467_2021_23730_MOESM1_ESM.pdf]

Supplementary information

Critical angle reflection imaging for quantification of molecular interactions on glass surface

Ma et al.

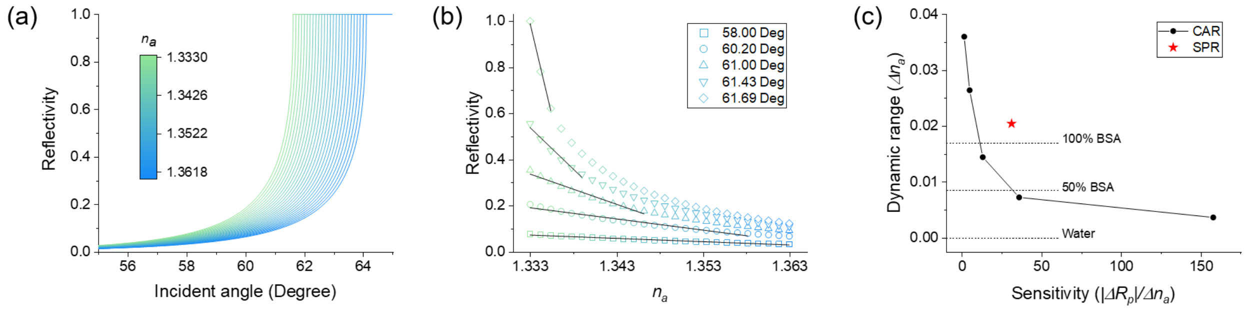

Supplementary Figure 1. Simulation results of CAR. (a) Relationship between reflectivity and incident angle at different aqueous solution refractive indices (n_a). (b) Reflectivity vs. n_a at five representative incident angles. The black lines are fittings of the linear regions ($R^2 > 0.97$). (c) Sensitivity and dynamic range of CAR at the five representative angles (black dots). Sensitivity and dynamic range are determined by the slope and the linear range of the black lines in (b). The red star marks the theoretical sensitivity and dynamic range of SPR. The simulation is shown in Supplementary Figure 2.

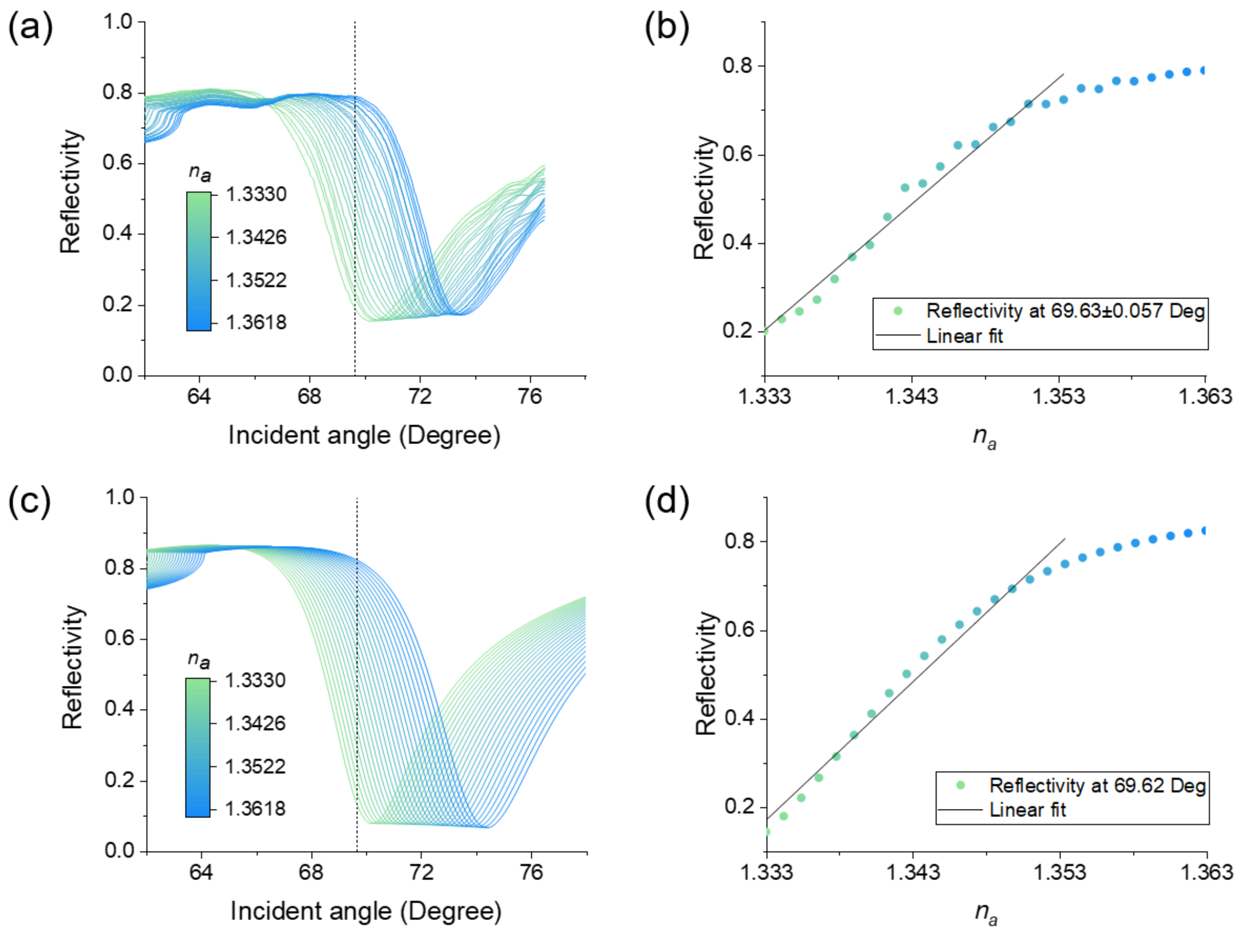

Supplementary Figure 2. Experimental and simulated SPR responses. (a) Measured reflectivity as a function of incident angle at different aqueous solution refractive indices (n_a). (b) Reflectivity vs. n_a at 69.63 degrees (marked by the vertical dash line in a). The black line is fitting of the linear region ($R^2 > 0.97$). The sensitivity and the dynamic range of SPR are determined by the slope and the linear range of the black line. (c) Simulated SPR reflectivity at different incident angles and n_a. (d) Reflectivity vs. n_a plot at 69.62 degrees (marked by the vertical dash line in c) obtained from the simulated data in c. The black line is fitting of the linear region ($R^2 > 0.97$), from which the theoretical sensitivity and dynamic range are calculated.

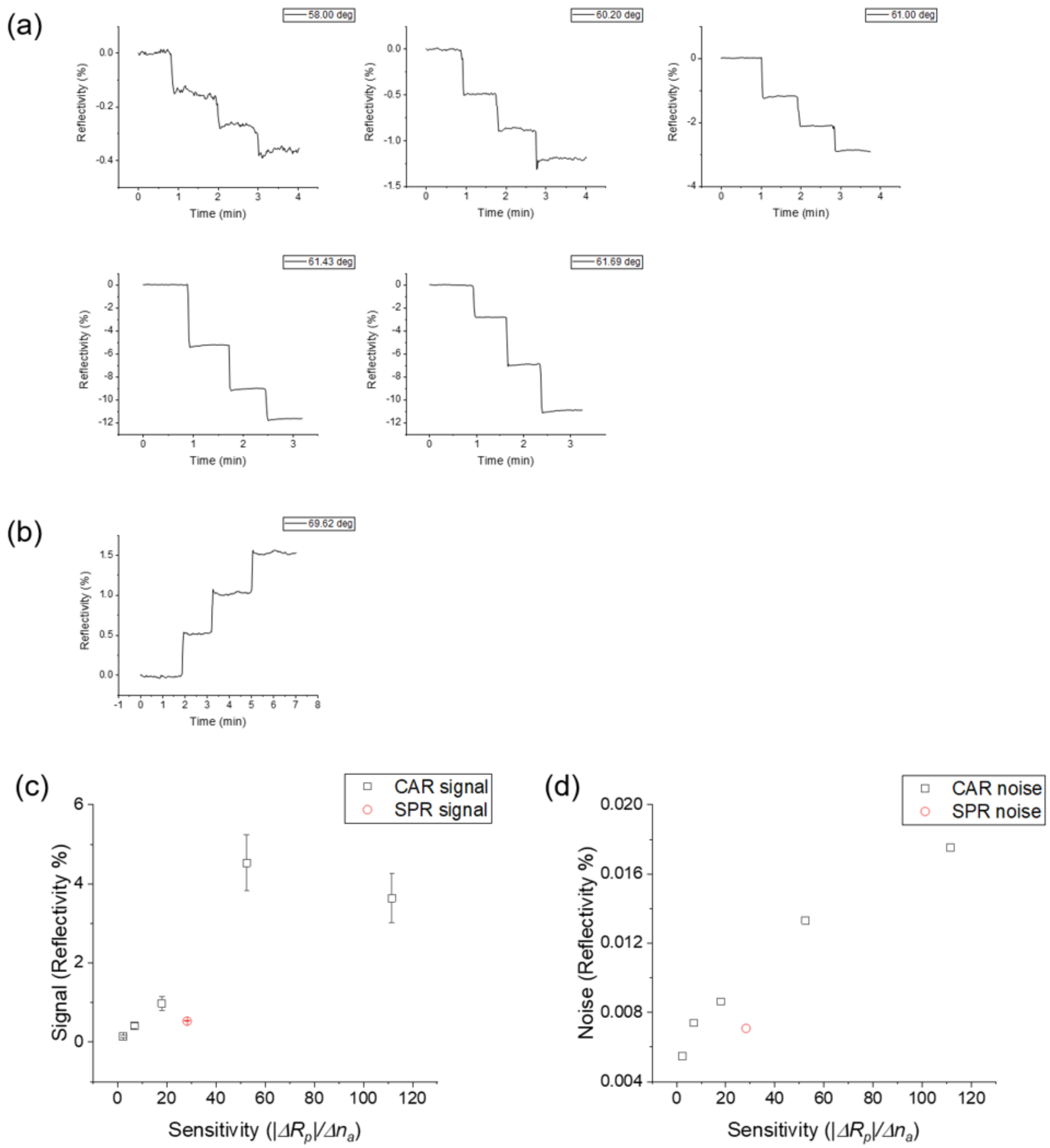

Supplementary Figure 3. Measuring the SNR for CAR and SPR. (a) Determining the SNR for CAR at five representative angles. 1% ethanol (final concentration) is added to water for three times, which leads to three reflectivity drops. The average value of the three responses is defined as the signal, and the standard deviation of the baseline (over 1 minute) is defined as the noise. (b) Determining the SNR for SPR. 1% ethanol (final concentration) is added to water for three times. The signal and noise are calculated the same way as in (a). (c) Signal of CAR and SPR. The dots and error bars represent mean and standard deviation obtained from three measurements. (d) Noise of CAR and SPR.

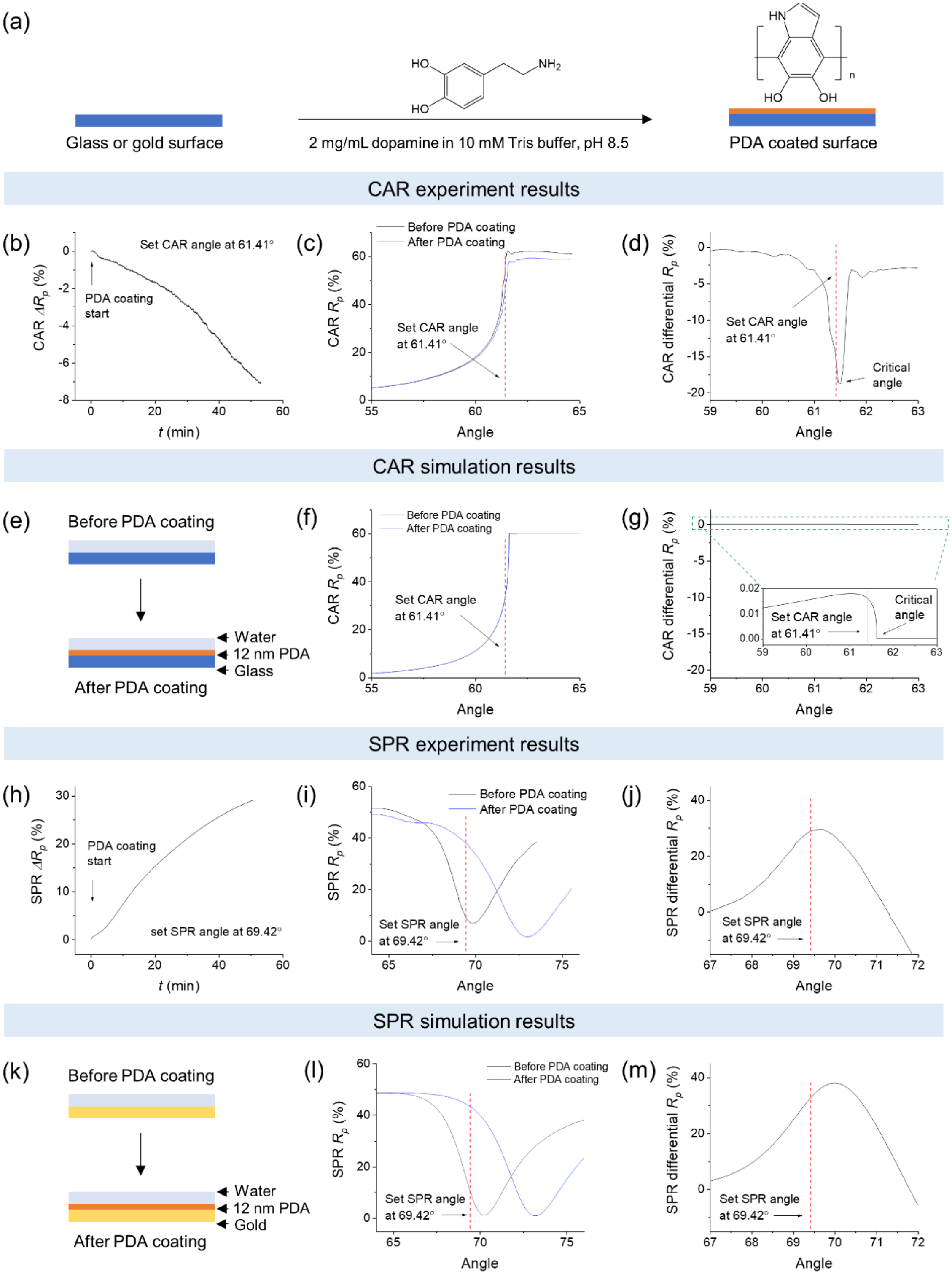

CAR vs. SPR (experiment results)

(n)

Substrate	CAR/SPR incident angle	PDA coating rate (nm/hr)	Response to PDA at 50 min (ΔR_p%)		Sensitivity (normalized to gold)	
			Real-time	End-point	Real-time	End-point
Glass	CAR at 61.41°	6 (ref 3)	7	15	0.54	1.2
Gold	SPR at 69.42°	14	30	30	1	1

Supplementary Figure 4. Sensitivity calibration for CAR and SPR using polydopamine (PDA). (a) The polymerization reaction of dopamine on glass or gold surface forms a thin layer of PDA.[1] (b) Real-time CAR response of PDA coating on glass surface. The CAR angle was set at 61.41°. For ease of comparison with SPR, the unit of CAR response is converted to reflectivity change (ΔR_p). ΔR_p is ~7% after 50 min of reaction. Note that the noise in the curve is caused by the Brownian motion of PDA nanoparticles formed in solution, because CAR has longer sensing distance than SPR and is more likely to pick up noises in solution. (c) End-point CAR measurement of PDA coating on glass. The R_p vs. incident angle curve was scanned before coating PDA (on bare glass) and after coating PDA for 50 min. For both measurements, pure Tris buffer (10 mM, pH 8.5, without dopamine) was used as medium to maintain the refractive index. The red dashed line marks the incident angle used for real-time measurement (same for the below figures). (d) Subtraction of the two curves in c shows the difference before and after PDA coating. At 61.41° (red dashed line), ΔR_p is ~15%. The end-point result is not consistent with the real-time result in b, because the real-time signal contains information both at the surface (PDA coating) and in the solution (PDA particle formation). In contrast, the end-point signal only contains signal at the surface. (e) Simulation of CAR response to 12 nm PDA layer on glass using Fresnel equation. Note that the refractive index of PDA is 1.55, as measured by Loget et al[2]. (f) CAR angle response curves for bare glass and glass with 12 nm PDA layer were simulated using Winspall, the two curves only show little difference (almost overlapped). (g) Subtraction of the two curves in f. The inset plot is zoom-in of the curve, which shows a different and much smaller response than that in d. This discrepancy implies the limitation of Fresnel equation in describing non-uniform thin layer reflectivity. (h) Real-time SPR response of PDA coating on gold surface. The SPR incident angle was set at 69.42°. The response after 50 min is 30% in R_p. (i) End-point SPR measurement of PDA coating. R_p vs. incident angle curve was measured before PDA coating and after coating PDA for 50 min. (j) Difference between the two curves in i. At 69.42° (red dashed line), ΔR_p is ~30%, consistent with the real-time result in h, which also indicates SPR is not sensitive to reactions in bulk solution. (k) Simulation of SPR response to 12 nm PDA layer on gold. (l) SPR angle response curves for bare gold and gold with 12 nm PDA layer were simulated using Winspall. (m) Difference between the two curves in l. The simulation results for SPR are close to the above experimental results in j. (n) Calculation of the relative sensitivity of CAR and SPR. The ΔR_p of CAR/SPR in response to 50 min of PDA coating are normalized by PDA coating rate on glass/gold. The coating rate on glass is obtained from literature[3], which is 6 nm/hour. The coating rate on gold is determined by SPR angle shift in figure i, which is 14 nm/hour, and is within the range reported in literature (7.8-19.5 nm/hour)[4, 5]. Sensitivity for CAR and SPR are calculated using both real-time and end-point responses. The result shows that the sensitivity ratio between CAR (at 61.41°) and SPR is 0.54 to 1.2, slightly lower than that from the ethanol calibration in Figure 1 (which has a ratio of 2). This ratio can reach a maximum of 2.3 at CAR angle of ~61.5°, but still lower than the ratio of 5 as determined by ethanol calibration in Figure 1d. This discrepancy suggests that other optical effects, such as scattering and interference, may contribute to CAR signal, in addition to the reflection model described by Fresnel equation.

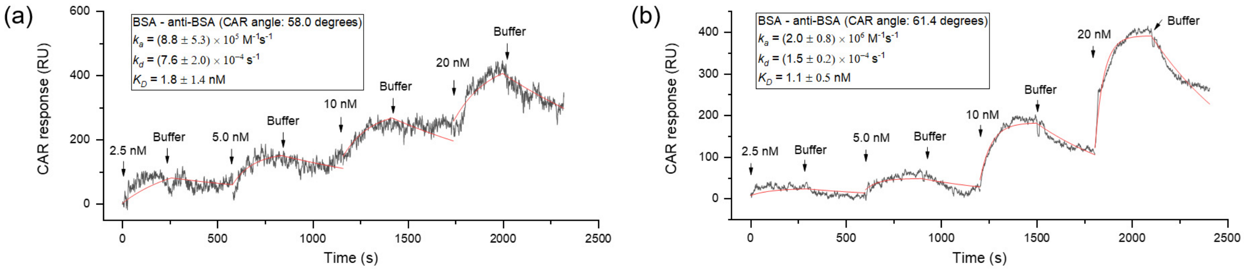

Supplementary Figure 5. Measuring anti-BSA binding to BSA at different CAR incident angles. The incident angle was set at (a) low angle (58.0 degrees) and (b) high angle (61.4 degrees).

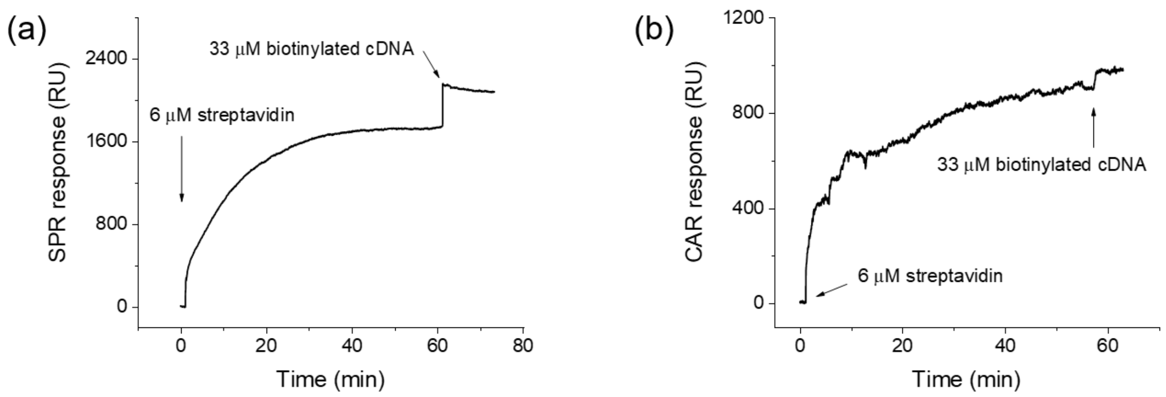

SPR
Sensitivity: $\frac{|\Delta R_p|}{\Delta n_a} \approx 25$
Streptavidin coverage: 1.7×10^{10} molecules/mm^2 (63%)
cDNA coverage: 2.5×10^{10} molecules/mm^2

CAR @ ~61.4 degrees
Sensitivity: $\frac{|\Delta R_p|}{\Delta n_a} \approx 50$
Streptavidin coverage: 9.0×10^{9} molecules/mm^2 (33%)
cDNA coverage: 7.0×10^{9} molecules/mm^2

Supplementary Figure 6. Surface coverage of streptavidin and cDNA measured with SPR and CAR. (a) An NHS/EDC activated gold surface was placed on the SPR setup. 6 μM streptavidin was flowed to the surface to induce immobilization of the streptavidin. Then 33 μM biotinylated cDNA was introduced which bound to the surface via biotin-streptavidin conjugation. (b) Immobilization of streptavidin on an epoxy activated glass surface followed by cDNA conjugation. The concentrations of streptavidin and cDNA are the same as (a). CAR angle was parked at ~61.4 degrees with a medium-high sensitivity. Note that the fluctuations between 5-12 minutes are due to the floating impurities in solution.

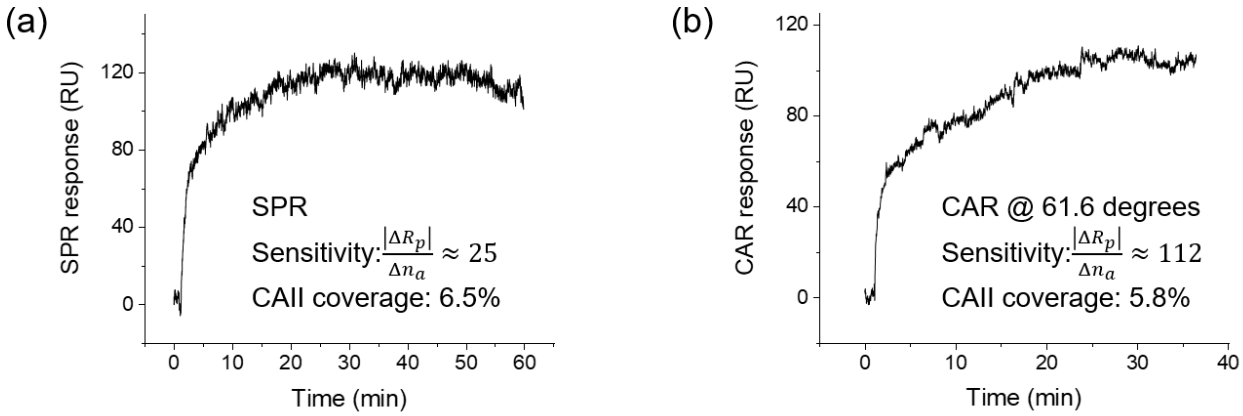

Supplementary Figure 7. Surface coverage of CAII on gold and glass surfaces. CAII immobilization process is monitored by SPR (a) and CAR (b). For CAR measurement, the incident angle is parked at 61.6 degrees, which has a sensitivity of ~112 RIU^{-1}. The coverage is estimated to be 6.5% and 5.8% on gold and glass surfaces, respectively. 6 nm is used as the diameter of CAII molecules for the coverage estimation.

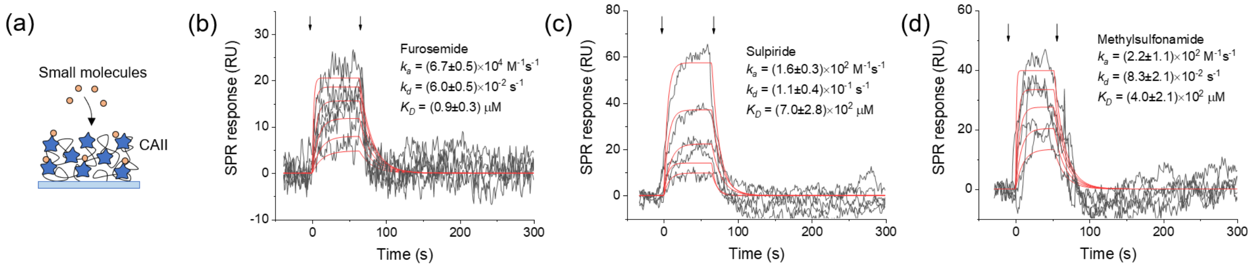

Supplementary Figure 8. Measuring small molecules binding to CAII on dextran coated gold surface with SPR. (a) CAII was immobilized on the dextran using NHS/EDC chemistry. Furosemide (331 Da), sulpiride (341 Da), and methylsulfonamide (95 Da), were flowed over the CAII functionalized surface. (b-d) SPR sensor response curves (black) and fittings (red) for the three small molecules. Furosemide concentrations: 238 nM, 475 nM, 938 nM, 1.88 μM, 3.75 μM, and 7.50 μM; Sulpiride concentrations: 62.5 μM, 125 μM, 250 μM, 500 μM, and 1.00 mM; Methylsulfonamide concentrations: 78.0 μM, 156 μM, 312 μM, 625 μM, and 2.50 mM. Note that the noise level of our result is higher than that in reference 4, which is due to instrument difference. Our SPR instrument is designed for imaging purpose, which does not have a reference flow channel for drift correction.

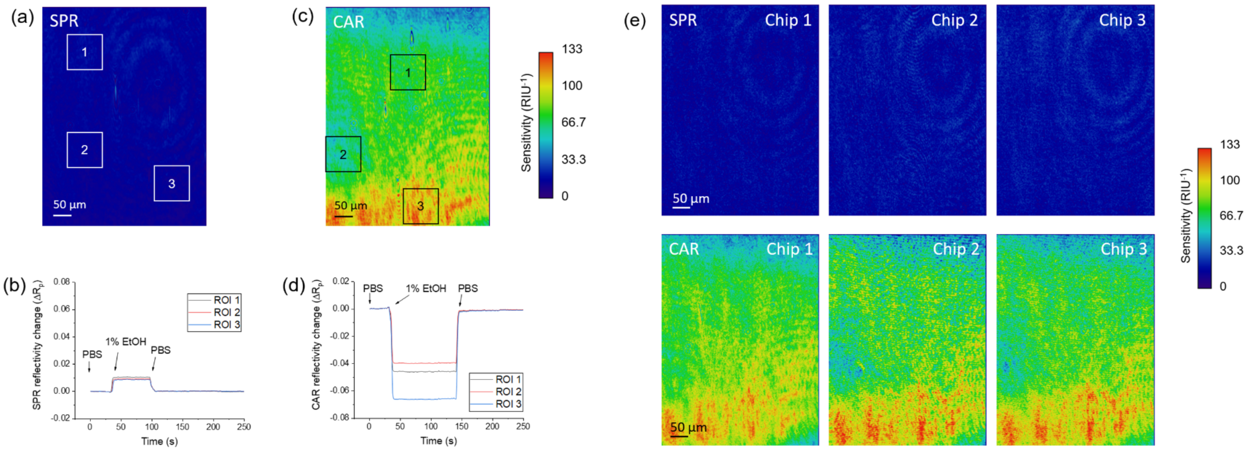

Supplementary Figure 9. Surface sensitivity distribution of CAR and SPR. (a) Spatial distribution of SPR sensitivity obtained by subtracting the images before and after 1% ethanol (final concentration) injection. (b) SPR response (reflectivity change) of the three regions of interests (ROIs) upon 1% ethanol injection. (c) CAR sensitivity is highly dependent on the incident angle. The angle was parked at ~61.5 degrees. The image shows the difference between before and after 1% ethanol injection, from which the sensitivity is calculated. The spatial distribution of sensitivity is non-uniform in vertical direction due to the slight difference in incident angle. (d) CAR response (reflectivity change) of the three ROIs upon 1% ethanol injection. The autosampler including the sample loop has a diffusion delay of less than 10 seconds. (e) Repeatability of the spatial sensitivity distribution. The experiments in (a) and (c) were repeated three times respectively using three different sensor chips and similar patterns were observed, implying the non-uniformity was due to instrument rather than sample.

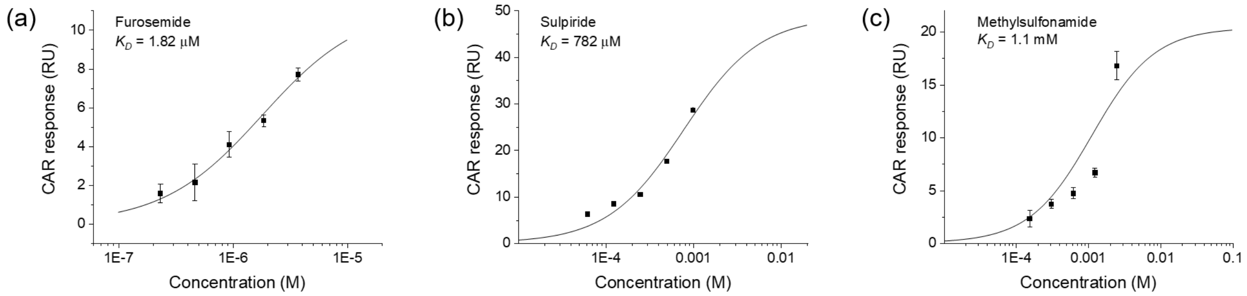

Supplementary Figure 10. Equilibrium analysis of furosemide, sulpiride and methylsulfonamide binding to CAII. The dissociation constants (K_D) are determined by fitting of the data (solid lines), which are 1.82 μM, 782 μM and 1.10 mM, respectively. The dots and error bars represent mean value and standard deviation of the maximum response for each concentration in Figures 3b-d.

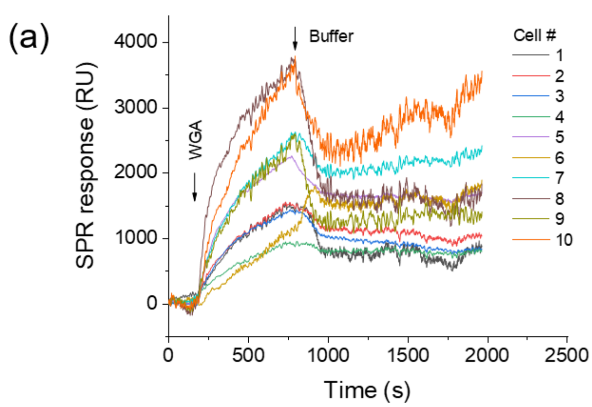

Supplementary Figure 11. WGA binding curves of individual fixed HeLa cells measured by SPR (a) and CAR (b).

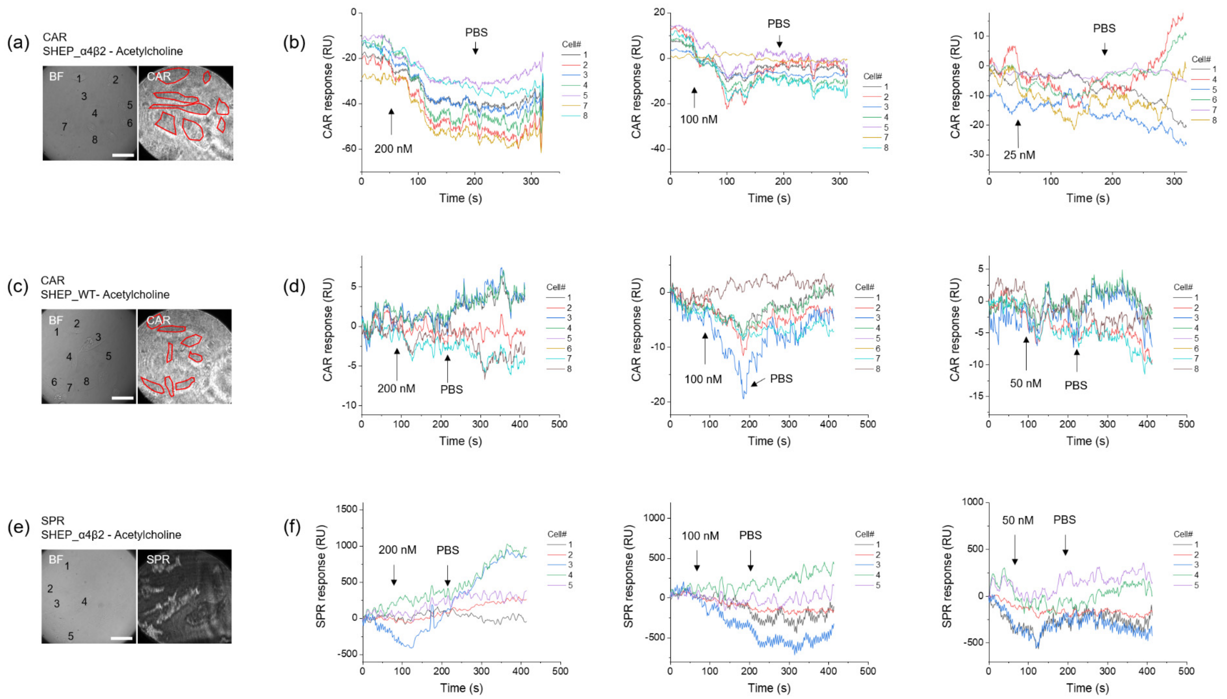

Supplementary Figure 12. Measuring acetylcholine-nAChR interaction with CAR and SPR. (a) Bright field (BF) and CAR images of 8 SH-EP1_α4β2 cells. The incident angle of CAR was parked at 61.6 degrees with high sensitivity of 112 RIU^{-1}. Because the angle was close to the critical angle, the cells (marked in red) only had little difference from the glass background which already reached critical angle. (b) CAR response of the cells upon flowing 200 nM (left), 100 nM (middle) and 25 nM (right) acetylcholine. (c) Control experiments: BF and CAR images of 8 wild type SH-EP1 cells. (d) CAR response of the cells upon flowing 200 nM (left), 100 nM (middle) and 50 nM (right) acetylcholine. (e) Measuring acetylcholine binding with SPR. BF and SPR images of 5 SH-EP1_α4β2 cells. (f) SPR response of the cells upon flowing 200 nM (left), 100 nM (middle) and 50 nM (right) acetylcholine. All scale bars are 50 μm. The above measurements were performed again using new sensor chips and cells, which showed similar results.

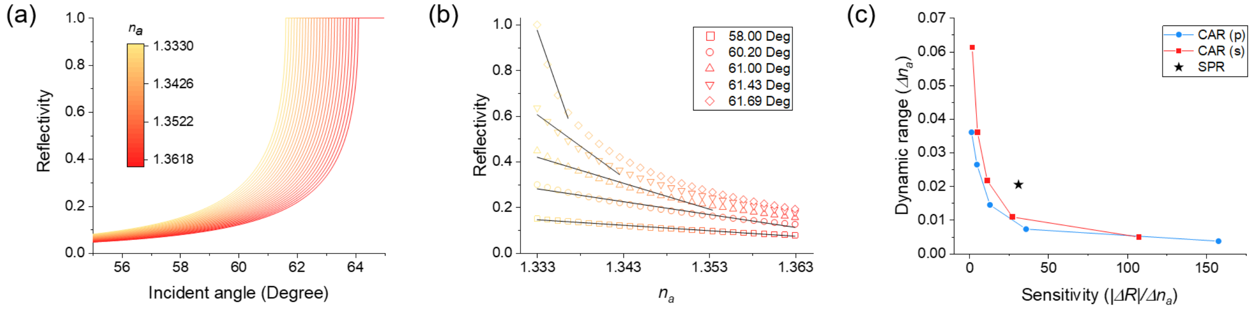

Supplementary Figure 13. Simulation results of CAR with *s*-polarized incident light. (a) Relationship between reflectivity and incident angle at different aqueous solution refractive indices (n_a). (b) Reflectivity vs. n_a at five representative incident angles. The black lines are fittings of the linear regions ($R^2 > 0.97$). (c) Sensitivity and dynamic range of CAR with *s*-polarization (CAR(s)) and *p*-polarization (CAR(p)) at the five representative angles. The star marks the theoretical sensitivity and dynamic range of SPR. The CAR(p) and SPR data are adopted from Supplementary Figure 1c.

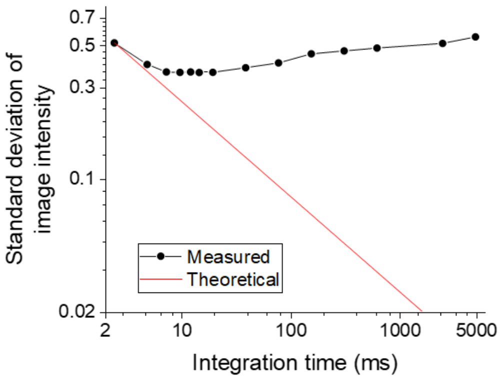

Supplementary Figure 14. System noise analysis. Total noise (black) and shot noise (red) were calculated using a method by Piliarik et al.[6] An image sequence was recorded at 500 frames per second for 1 min. The images were averaged over different periods and the new image sequences were differentiated. Standard deviation of the differentiated image sequence was calculated and plotted vs. the integration time. At 1 s which is the typical sampling rate for biosensors, the total noise is 25 times larger than the shot noise.

Reference

1. Lee, H., Dellatore, S.M., Miller, W.M. & Messersmith, P.B. Mussel-Inspired Surface Chemistry for Multifunctional Coatings. *Science* **318**, 426-430 (2007).
2. Loget, G., Wood, J.B., Cho, K., Halpern, A.R. & Corn, R.M. Electrodeposition of Polydopamine Thin Films for DNA Patterning and Microarrays. *Analytical Chemistry* **85**, 9991-9995 (2013).
3. Terrill, H.C. Optimization of Polydopamine Coatings. (2015).
4. Wood, J.B., Szyndler, M.W., Halpern, A.R., Cho, K. & Corn, R.M. Fabrication of DNA microarrays on polydopamine-modified gold thin films for SPR imaging measurements. *Langmuir* **29**, 10868-10873 (2013).
5. Ribena, D. Dopamine modification of interfaces between polymers and metals. (2012).
6. Piliarik, M. & Sandoghdar, V. Direct optical sensing of single unlabelled proteins and super-resolution imaging of their binding sites. *Nature Communications* **5**, 4495 (2014).
