# Supplementary material for: Critical angle reflection imaging for quantification of molecular interactions on glass surface
Source: Nat Commun. 2021 Jun 7;12:3365. doi: 10.1038/s41467-021-23730-8 (PMC8185113; doi:10.1038/s41467-021-23730-8)
Supplement: Supplementary file 3 — Description of Additional Supplementary Files [file 41467_2021_23730_MOESM3_ESM.docx]

Description of Additional Supplementary Files

Title: Supplementary Movie 1.

Description: SPR response of glycoprotein-WGA binding. The cell is number 10 in Figure 4b. The video shows the association phase.

Title: Supplementary Movie 2.

Description: CAR response of glycoprotein-WGA binding. The cell is number 7 in Figure 4e. The video shows the association phase.

Title: Supplementary Movie 3.

Description: Differential CAR images of a live cell. The cell is the same one as shown in Figure 6a.
