# Supplementary material for: Critical angle reflection imaging for quantification of molecular interactions on glass surface
Source: Nat Commun. 2021 Jun 7;12:3365. doi: 10.1038/s41467-021-23730-8 (PMC8185113; doi:10.1038/s41467-021-23730-8)
Supplement: Supplementary file 7 — Reporting Summary [file 41467_2021_23730_MOESM7_ESM.pdf]

nature research

Corresponding author(s): Shaopeng Wang
Last updated by author(s): Apr 23, 2021

Reporting Summary

Nature Research wishes to improve the reproducibility of the work that we publish. This form provides structure for consistency and transparency in reporting. For further information on Nature Research policies, see our Editorial Policies and the Editorial Policy Checklist.

Statistics

For all statistical analyses, confirm that the following items are present in the figure legend, table legend, main text, or Methods section.

n/a	Confirmed	
☐	☒	The exact sample size (*n*) for each experimental group/condition, given as a discrete number and unit of measurement
☐	☒	A statement on whether measurements were taken from distinct samples or whether the same sample was measured repeatedly
☒	☐	The statistical test(s) used AND whether they are one- or two-sided<br>*Only common tests should be described solely by name; describe more complex techniques in the Methods section.*
☒	☐	A description of all covariates tested
☒	☐	A description of any assumptions or corrections, such as tests of normality and adjustment for multiple comparisons
☐	☒	A full description of the statistical parameters including central tendency (e.g. means) or other basic estimates (e.g. regression coefficient) AND variation (e.g. standard deviation) or associated estimates of uncertainty (e.g. confidence intervals)
☒	☐	For null hypothesis testing, the test statistic (e.g. *F*, *t*, *r*) with confidence intervals, effect sizes, degrees of freedom and *P* value noted<br>*Give P values as exact values whenever suitable.*
☒	☐	For Bayesian analysis, information on the choice of priors and Markov chain Monte Carlo settings
☒	☐	For hierarchical and complex designs, identification of the appropriate level for tests and full reporting of outcomes
☒	☐	Estimates of effect sizes (e.g. Cohen's *d*, Pearson's *r*), indicating how they were calculated

Our web collection on statistics for biologists contains articles on many of the points above.

Software and code

Policy information about availability of computer code

Data collection	Images obtained on the prism-based setup were recorded using ImageSPR Version 1.8.0.5 (Biosensing Instrument Inc.). For experiments conducted on the microscope-based setup, the images were collected using HCImageLive Version 4.4.5.0.
Data analysis	Images were analyzed using Fiji v1.52p and ImageAnalysis Version 1.8.0.5 (Biosensing Instrument Inc.). ImageAnalysis and Scrubber 2 were used to fit the binding kinetics curves. Winspall Version 3.02 was used to simulate the reflectivity of SPR and CAR. All data were plotted using Origin 2019 and MATLAB R2019a.

For manuscripts utilizing custom algorithms or software that are central to the research but not yet described in published literature, software must be made available to editors and reviewers. We strongly encourage code deposition in a community repository (e.g. GitHub). See the Nature Research guidelines for submitting code & software for further information.

Data

Policy information about availability of data

All manuscripts must include a data availability statement. This statement should provide the following information, where applicable:

- Accession codes, unique identifiers, or web links for publicly available datasets
- A list of figures that have associated raw data
- A description of any restrictions on data availability

The data that support the findings of this study are available in Dryad Digital Repository (DOI: 10.5061/dryad.47d7wm3d0).

April 2020

Field-specific reporting

Please select the one below that is the best fit for your research. If you are not sure, read the appropriate sections before making your selection.

- [x] Life sciences
- [ ] Behavioural & social sciences
- [ ] Ecological, evolutionary & environmental sciences

For a reference copy of the document with all sections, see nature.com/documents/nr-reporting-summary-flat.pdf

Life sciences study design

All studies must disclose on these points even when the disclosure is negative.

Sample size	No sample size determination was performed. Sample size was deemed sufficient when binding kinetics can be determined from fitting of the data. For the majority of the studies, each sample was measured at 3-5 different concentrations to extract the kinetics.
Data exclusions	About 20% of the measurements failed due to instability of the fluidics in CAR and SPR systems. These failures can be easily identified by eye during the measurement, as they generate huge jump or drift in the sensorgram.
Replication	At least two replicates were measured for major findings, including sensitivity calibration, biomolecular interactions, small molecule-protein interactions, and cell-ligand interactions to confirm reproducibility. Each replicate was measured on a new sensor chip with samples prepared from different batches. All attempts at replication were successful.
Randomization	The cells chosen for ligand binding measurement were randomly selected. Randomization was not relevant to the CAR and SPR measurements, because the type and concentration of the binding pairs were given before the measurements.
Blinding	The investigator was not blinded to group allocation during the experiments.

Reporting for specific materials, systems and methods

We require information from authors about some types of materials, experimental systems and methods used in many studies. Here, indicate whether each material, system or method listed is relevant to your study. If you are not sure if a list item applies to your research, read the appropriate section before selecting a response.

Materials & experimental systems

n/a	Involved in the study
[ ]	[x] Antibodies
[ ]	[x] Eukaryotic cell lines
[x]	[ ] Palaeontology and archaeology
[x]	[ ] Animals and other organisms
[x]	[ ] Human research participants
[x]	[ ] Clinical data
[x]	[ ] Dual use research of concern

Methods

n/a	Involved in the study
[x]	[ ] ChIP-seq
[x]	[ ] Flow cytometry
[x]	[ ] MRI-based neuroimaging

Antibodies

Antibodies used	Mouse anti-cattle BSA monoclonal antibody was purchased from MyBioSource (Catalog number: MBS7135229).
Validation	The antibody was validated by Western Blot by the manufacturer. Our binding kinetics measurements (Figures 2b-c) serve as an additional validation of the antibody.

Eukaryotic cell lines

Policy information about cell lines

Cell line source(s)	HeLa and SH-EP1 cells were purchased from the American Type Culture Collection. SH-EP1_α4β2 was provided as a gift from Dr. Jie Wu at Barrow Neurological Institute (Dr. Wu is now a faculty at University of Arizona). A reference is provided in the manuscript on the generation of the cell line: DOI: 10.1074/jbc.M400335200
Authentication	None of the cell lines used were authenticated.
Mycoplasma contamination	The cell lines were not tested for mycoplasma contamination.
Commonly misidentified lines (See ICLAC register)	No commonly misidentified cell lines were used in this study.
